# Vitamin D receptor expression controls proliferation of naïve CD8^+^ T cells and development of CD8 mediated gastrointestinal inflammation

**DOI:** 10.1186/1471-2172-15-6

**Published:** 2014-02-07

**Authors:** Jing Chen, Danny Bruce, Margherita T Cantorna

**Affiliations:** 1 Department of Veterinary and Biomedical Science, The Pennsylvania State University, University Park, PA 16802, USA; 2 Center for Molecular Immunology and Infectious Disease, The Pennsylvania State University, University Park, PA 16802, USA; 3 Pathobiology Graduate Program, The Pennsylvania State University, University Park, PA 16802, USA; 4 Present address: Lineberger Comprehensive Cancer Center, School of Medicine, University of North Carolina, Chapel Hill, NC 27599, USA; 5 Department of Veterinary and Biomedical Sciences, The Center for Molecular Immunology and Infectious Disease, 115 Henning Bldg, University Park, PA 16802, USA

**Keywords:** Vitamin D receptor, CD8^+^ T cells, Proliferation, Inflammatory bowel disease

## Abstract

**Background:**

Vitamin D receptor (VDR) deficiency contributes to the development of experimental inflammatory bowel disease (IBD) in several different models. T cells have been shown to express the VDR, and T cells are targets of vitamin D. In this article we determined the effects of VDR expression on CD8^+^ T cells.

**Results:**

VDR KO CD8^+^ T cells, but not WT CD8^+^ T cells, induced colitis in Rag KO recipients. In addition, co-transfer of VDR KO CD8^+^ T cells with naïve CD4^+^ T cells accelerated colitis development. The more severe colitis was associated with rapidly proliferating naïve VDR KO CD8^+^ T cells and increased IFN-γ and IL-17 in the gut. VDR KO CD8^+^ T cells proliferated *in vitro* without antigen stimulation and did not downregulate CD62L and upregulate CD44 markers following proliferation that normally occurred in WT CD8^+^ T cells. The increased proliferation of VDR KO CD8^+^ cells was due in part to the higher production and response of the VDR KO cells to IL-2.

**Conclusions:**

Our data indicate that expression of the VDR is required to prevent replication of quiescent CD8^+^ T cells. The inability to signal through the VDR resulted in the generation of pathogenic CD8^+^ T cells from rapidly proliferating cells that contributed to the development of IBD.

## Background

Inflammatory bowel diseases (IBD) are immune mediated diseases that result because of a complex interaction between genetics and the environment. Over 3.6 million people in the US and Europe suffer from IBD. Environmental factors that contribute to IBD development include the composition of the normal bacterial flora [1] and perhaps vitamin D. A major source of vitamin D results from its manufacture via a photolysis reaction in the skin and vitamin D available from sunlight exposure is significantly less in northern climates, and especially low during the winter [2]. In addition dietary intake of vitamin D is problematic since there are few foods, which are naturally rich in vitamin D. There is evidence for a link between vitamin D availability and the prevalence of immune mediated diseases in general and IBD in particular [3,4]. In addition, experimental models of IBD are more severe in vitamin D receptor (VDR) KO and vitamin D deficient models [5]. Vitamin D is likely an environmental factor that affects the development of IBD.

T cells have been shown to express the VDR, and T cells are both direct and indirect targets of vitamin D. In addition, the active form of vitamin D (1,25(OH)_2_D_3_) suppressed the development of experimental models of T cell mediated diseases including IBD, multiple sclerosis and type-1 diabetes [6,7]. Conversely, vitamin D deficiency and/or VDR deficiency resulted in an exacerbation of experimental IBD [5]. Expression of the VDR does not affect the development of normal numbers of CD4 and CD8αβ T cells in the thymus or in the periphery [5,8,9]. VDR KO CD4^+^ T cells express more IL-17, and IFN-γ, proliferate more rapidly in a mixed lymphocyte reaction and induce more severe colitis than WT CD4 cells [5,10]. Mice with VDR specific KO in T cells have increased incidence of experimental autoimmune encephalomyelitis and VDR expression in T cells was shown to be essential for the effectiveness of 1,25(OH)_2_D_3_ for the suppression of disease [11]. 1,25(OH)_2_D_3_*in vitro* suppressed the proliferation of both CD4^+^ and CD8^+^ T cells and inhibited the production of IFN-γ, and IL-2 [12,13]. Vitamin D is required for the development of two regulatory cell populations: NKT cells and CD8αα expressing T cells [9,14]. In addition, 1,25(OH)_2_D_3_ induces CD4^+^ T regulatory cells *in vitro* and *in vivo*[15,16]. Resting and activated CD8^+^ T cells have been shown to express higher levels of the VDR than CD4^+^ T cells; however, the physiological role of vitamin D and the VDR in regulating CD8^+^ T cell function has not been examined [17].

CD8^+^ T cells can be pathogenic, protective or tolerogenic. Pathogenic and protective CD8^+^ T cells share common features including IFN-γ and TNF-α production as well as cytotoxicity [18,19]. A pathogenic role for CD8^+^ T cells has been shown for IBD [18]. Heat shock protein-specific CD8^+^ T cells transferred severe symptoms of IBD in immunodeficient mice due to the production of IFN-γ and TNF-α [18]. Conversely, several CD8^+^ T cell subsets have been shown to act in a suppressive or regulatory manner. CD8^+^ T cells that do not express CD28 (CD8/CD28^−^), but produced IL-10 in human peripheral blood, suppressed cytotoxic activity and proliferation *in vitro*[20]. In mice, IL-10 and TGF-β producing CD8/CD28^−^ regulatory T cells inhibited experimental IBD development in the T cell transfer model of IBD [21]. Human CD8/CXCR3^+^ cells are a second population of human regulatory CD8^+^ T cells [22]. Like the CD8/CD28^−^ T cells, the mouse homologs of the human CD8/CXCR3^+^ T cells (CD8/CD122^+^) suppressed the development of T cell-transfer colitis via the production of IL-10 [23]. Lastly, CD8αα^+^TCRαβ^+^ cells found in the intraepithelial lymphocytes (IEL) of the small intestine (SI) have IL-10 mediated regulatory abilities and can suppress development of T cell-transfer colitis [24,25]. In experimental IBD CD8^+^ T cells can either cause or protect from disease development.

Here we determined the effects of vitamin D on CD8αβ^+^ T cells. VDR KO mice had increased numbers of naïve CD8^+^ T cells that when purified and then transferred to Rag KO recipients induced colitis as determined by histological staining. In addition, co-transfer of VDR KO CD8^+^ T cells with naïve WT CD4^+^ cells accelerated the development of colitis in Rag KO recipients. The cause of the VDR KO CD8^+^ T cell pathogenicity was due to the uncontrolled proliferation of CD8^+^ T cells in general and naïve CD8^+^ T cells in particular. In addition, VDR KO CD8^+^ T cells overproduced IL-2. The rapidly dividing VDR KO CD8^+^ T cells accumulated in the gut where they induced IFN-γ and IL-17 production.

## Methods

### Mice

WT, VDR KO, IL-10 KO, IL-10/VDR double (D) KO and Rag KO mice all on the C57BL/6 background were bred in the animal facilities at the Pennsylvania State University (University Park, PA). The original VDR KO breeders were a gift from Dr. Marie Demay (Harvard University, Boston MA) and the DKO mice were generated as previously described [5]. All other breeders were originally from Jackson Laboratories (Bar Harbor, ME). Experimental procedures were approved by the Office of Research Protection Institutional Animal Care and Use Committee at the Pennsylvania State University.

### T cell isolation

CD4^+^ or CD8^+^ T cells were purified from the spleen using the mouse CD4^+^ or CD8^+^ Recovery Column kit and the manufacturer’s instructions (Cedarlane, Burlington, NC). Column purification was followed by cell sorting (Cytopeia Influx, Seattle, WA) of CD4^+^, CD8^+^, CD4^+^CD45RB^high^, CD8^+^CD28^+/-^, CD8^+^CD122^+^ T cell subsets. Post-sorting analysis confirmed that the purity of the T cells was >99% (Additional file 1: Figure S1A). IELs were isolated for analyses by removing the Peyer’s patches and splitting the SI lengthwise followed by cutting the SI into 0.5 cm pieces. The pieces were incubated twice in HBSS containing 0.15 μg/ml dithiothreitol and 5%FBS (Sigma-Aldrich, St. Louis, MO) for 20 min at 37°C under 200 rpm rotation. The IEL were collected from the interface of 40/80% Percoll gradients (Sigma-Aldrich).

### Adoptive transfer

All transfers used T cells from the spleens of the donor mice. Groups of Rag KO mice were injected intraperitoneally (i.p.) with 1 × 10^6^ CD8^+^ cells or CD8 T cell sorted subsets (CD28^−^, CD28^+^ or CD122^+^) from WT, VDR KO, IL-10 KO or IL-10/VDR DKO mice. Sorting purity and gating strategies are in Additional file 1: Figure S1. For the co-transfer experiments, Rag KO mice were injected i.p. with 1 × 10^6^ WT or VDR KO (CD45.2^+^) CD8^+^ T cells on day -1 and 4 × 10^5^ WT (CD45.1^+^) CD4^+^CD45RB^high^ cells on day 0. Both CD8 and CD4^+^CD45RB^high^ cells were resuspended in 200 μL PBS. The Rag KO recipients were weighed weekly and euthanized after 7 or 8 wks.

### Colitis development

Colitis symptoms measured included: weight loss, colon/body weight (BW) ratios, histopathology scores, diarrhea, rectal bleeding, and rectal prolapse exactly as described [5]. 1 cm of the distal colon was fixed in formalin and sent to the Penn State University Animal Diagnostic Laboratories (University Park, PA) for sectioning and haematoxylin & eosin (H&E) staining. Inflammation and epithelial injury of the colons were scored blindly by two individuals for inflammation (0-4) and epithelial hyperplasia (0-4) [5]. Total histopathology scores ranged from 0-8.

### BrdU incorporation assay

Rag KO mice were injected i.p. with 50 μl of 25 mg/ml BrdU dissolved in PBS every two days. The mice were euthanized at d3, d8 and d14 and the MLN and IEL were fixed with ice-cold 95% ethanol and paraformaldehyde digested with DNaseI solution (Sigma).

### Flow cytometry

Cells were stained with: FITC anti-CD8α, FITC anti-CD8β, FITC anti-CD45RB, PE anti-CD45.1, PE anti-CD28, PE anti-CD122, PE anti-CD4, PE anti-CD44, PE anti-CD25, PE-Texas Red (ECD) anti-CD4, PECy5 anti-CD62L, PECy5 anti-TCRβ and PECy7 anti-CD8α (BD Pharmingen, San Jose, CA) for surface markers. Gating strategies for FACS analysis are shown in Additional file 2: Figure S2. For BrdU incorporation assays, cells were stained with Biotin anti-BrdU, FITC streptavidin and Biotin Mouse IgG1, κ isotype control (Biolegend, San Diego, CA). For intracellular cytokine staining, IEL were stimulated with PMA (0.1 μg/ml, Sigma), ionomycin (0.5 μg/ml, Sigma) and Brefeldin A (10 μg/ml, Sigma) for 6 h, fixed with 4% paraformadehyde (Sigma-Aldrich), permeabilized with 0.1% saponin (Sigma-Aldrich), and stained with FITC anti-IFNγ, PE anti-IL-17A, or the FITC/PE labeled Rat IgG1 isotype controls (BD Pharmingen). Flow cytometry was done on a FC500 bench top cytometer (Beckman Coulter, Brea, CA) and the data was analyzed with FlowJo 7.6.5 software (TreeStar, Ashland, OR).

### Cell culture and CFSE staining

Purified CD8^+^ T cells were labeled with CFSE using Cell TRave CFSE Cell Proliferation Kit (Life Technologies, Grand Island, NY). The labeled CD8^+^ T cells (2 × 10^6^ cells/ml) were cultured with plate bound anti-CD3 (5 μg/ml) and anti-CD28 (5 μg/ml, BD Pharmingen) or 0.2 μg/ml recombinant IL-2 with/without neutralizing IL-2 antibodies (50 ng/ml, Clone: S4B6, BD Pharmingen). Supernatants were collected from 3d cultures of unstimulated or CD3/CD28 stimulated CD8^+^ T cells, and IL-2 in the supernatant was evaluated using an ELISA kit and the manufacturer’s instructions (BD Pharmingen).

### Quantitative real-time PCR

Total RNA was isolated from SI and colon of mice following the manufacturer’s instructions (Qiagen, Valencia, CA). cDNA was synthesized by using the TaqMan reverse transcription reagents kit (Applied Biosystems, Carlsbad, CA) and was amplified for cytokines *Ifn-γ, Il-17A* and *Il-10* with SYBR green mix (BioRad, Hercules, CA) by MyiQ Single-Color Real-Time PCR machine (BioRad). Expression levels of these cytokines were normalized by GAPDH and calculated by using ΔΔCt method [2^(Ct_sample_ –Ct_ctrl_)].

### Statistics

Statistical analyses were performed by GraphPad (PRISM software, La Jolla, CA). Data are presented as mean ± SEM values from two or three experiments. Unpaired Student’s *t* test, and ANOVAs with Bonferroni post-hoc tests were used to calculate statistical significance. Values are significantly different with *P*-values of *P < 0.05, **P < 0.01, and ***P < 0.001.

## Results

### CD8^+^ T cells from VDR KO mice transfer IBD to Rag KO recipients

**Figure 1:**
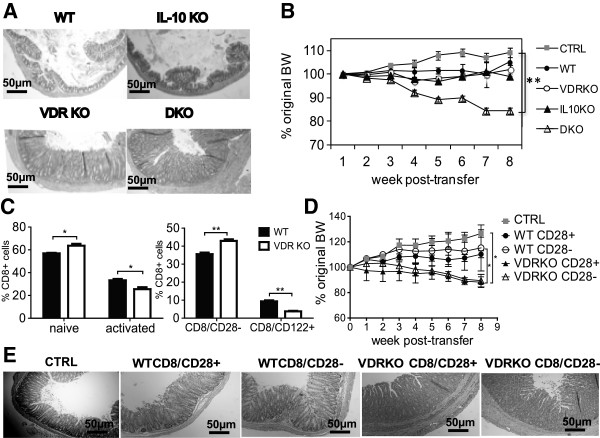
**CD8**^**+ **^**T cells from VDR KO mice induce colitis in Rag KO recipients.** Sorted CD8^+^ T cells from WT, IL-10 KO, VDR KO, and IL-10/VDR DKO mice were injected (10^6^ cells per mouse i.p.) into Rag KO recipients. CTRL had no cell transfer. **(A)** Representative colonic sections from the Rag KO recipients of CD8^+^ T cells 8 weeks post-transfer. Colonic samples were stained with H&E (scoring system in *Methods*) and were shown at 10× magnification; scale bar = 50 μm. Colon sections shown were rated: WT (score = 0), IL-10 KO (score = 0), VDR KO (score = 7), and DKO (score = 8). **(B)** The percentage change in BW of Rag KO recipients of CD8^+^ T cells from WT, IL-10 KO, VDR KO and DKO mice. **(C)** The phenotype of CD8^+^ T cells from the spleen of WT and VDR KO mice. The splenic lymphocytes were prepared and stained for TCRβ, CD8β, CD44, CD62L, CD28 and CD122 antibodies. The gating strategy is in Additional file 2: Figure S2A. **(D)** The percentage change in BW of the Rag KO recipients following transfer of sorted CD8^+^/CD28^+^ or CD8^+^/CD28^-^ cells from WT or VDR KO mice (10^6^ cells per mouse i.p.). **(E)** Representative H&E stained colon sections from Rag KO recipients of CD8^+^/CD28^+^ or CD8^+^/CD28^-^ T cells at 8 weeks post-transfer. Colon sections shown were rated: CTRL (score = 1), WT CD8/CD28^+^ (score = 3), WT CD8/CD28^−^ (score = 3), VDR KO CD8/CD28^+^ (score = 6), VDR KO CD8/CD28^−^ (score = 6). Values represent the mean ± SEM of 5-8 mice per group and one representative of three independent experiments (A-E). Two-way ANOVA (B,D) or Student’s t-tests (C), *P < 0.05, **P < 0.01.

CD8^+^ T cells from WT and IL-10 KO mice did not induce colitis when transferred to Rag KO recipients and histopathology of the colons from Rag KO mice that received 10^6^ WT or IL-10 KO CD8^+^ T cells were normal (Figure 1A). Conversely, Rag KO mice that received 10^6^ VDR KO or IL-10/VDR DKO CD8^+^ T cells showed severe inflammation in the colon including hyperplasia and infiltration of immune cells (Figure 1A). The Rag KO mice that received CD8^+^ T cells from DKO mice lost a significant amount of their starting BW by 8 wks (Figure 1B). In fact the Rag KO recipients of DKO CD8^+^ T cells developed a fulminating form of IBD that resembled the IBD that develops in the DKO mice [5]. Rag KO mice that received no cells (CTRL) gained weight over the 8 wk study and Rag KO mice that received WT, VDR KO and IL-10 KO CD8^+^ T cells maintained their BW (Figure 1B). None of the over 20 Rag KO recipients of VDR KO CD8^+^ T cells lost weight even though the mice had intense inflammation in both the colon and SI (data not shown and Figure 1A). The colon showed the greatest degree of inflammation following transfer of VDR KO CD8^+^ T cells to Rag KO recipients.

In order to determine what the differences were between VDR KO and WT CD8^+^ T cells, the CD8^+^ T cells were characterized from WT and VDR KO mice. There was a higher frequency of naïve (CD62L^high^/CD44^low^) and CD8/CD28^−^ cells and a lower frequency of activated (CD62L^low^/CD44^high^) and CD8/CD122^+^ cells in the spleen of VDR KO compared to WT mice (Figure 1C). CD25^+^ expression on CD8^+^ T cells was low and less than 2% of the CD8^+^ T cells in VDR KO and WT mice expressed CD25 (Additional file 1: Figure S1C). In addition, there was no difference between expression of CD25 on VDR KO and WT CD8^+^ T cells (Additional file 1: Figure S1C). There was no difference in the total number of splenocytes isolated from WT and VDR KO mice and therefore changes in frequency also resulted in changes in absolute numbers of cells. The frequency of granzyme B^+^ cells was not different in CD3/CD28 activated VDR KO and WT CD8^+^ T cells (data not shown). IL-17 and IL-10 were undetectable while IFN-γ was not different in the supernatants from CD3/CD28 activated VDR KO and WT CD8^+^ T cells (data not shown). The finding of more regulatory CD8/CD28^−^ and less regulatory CD8/CD122^+^ from the VDR KO mice was followed up by doing additional transfers to Rag KO mice using purified populations of these regulatory cell types.

Rag KO recipients of 10^6^ sorted WT CD8/CD28^−^ or CD8/CD28^+^ T cells weighed the same as the CTRL Rag KO mice that did not receive any cells (Figure 1D). Conversely, Rag KO recipients of 10^6^ sorted VDR KO CD8/CD28^−^ or CD8/CD28^+^ T cells lost significantly more weight than the CTRL and WT CD8 T cell recipients (Figure 1D). Sorted 10^6^ CD8/CD122^+^ T cell transfers from either WT or VDR KO mice did not induce weight loss or colitis in Rag KO recipients (data not shown). Histopathology of colonic tissue from Rag KO recipients of VDR KO CD8^+^ subsets (CD28^+/−^) showed significantly more inflammation and epithelial hyperplasia than CTRL (Figure 1E). Histopathology of colonic tissue from Rag KO recipients of WT CD8^+^ subsets (CD28^+/−^) was not different from CTRL (Figure 1E). Increased colitis in Rag KO recipients of VDR KO CD8^+^ T cells is not a result of a difference in the CD28^+/−^ subpopulations.

### VDR KO CD8^+^ T cells accelerate CD4/CD45RB^high^ cell-mediated colitis

**Figure 2:**
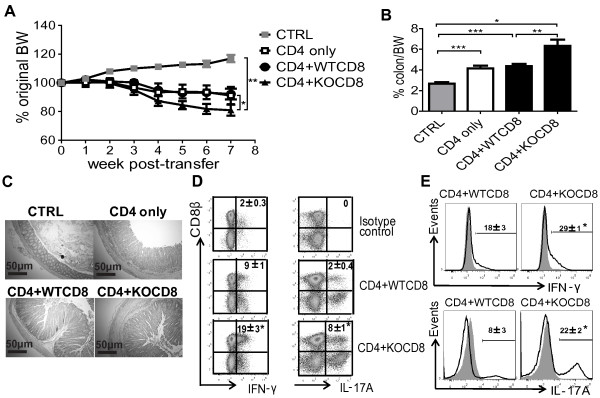
**VDR KO CD8**^**+ **^**T cells aggravate CD4/CD45RB**^**high **^**cell-induced colitis.** Rag KO mice were injected i.p. with sorted 10^6^ WT or VDR KO (CD45.2^+^) CD8^+^ T cells on day -1 and 4 × 10^5^ WT (CD45.1^+^) CD4^+^CD45RB^high^ cells on day 0. **(A)** The percentage change in original BW of Rag KO mice recipients of CTRL, or CD4/CD45RB^high^ (CD4 only), CD4/CD45RB^high^ plus WT CD8 (CD4 + WTCD8), CD4/CD45RB^high^ plus VDR KO CD8 (CD4 + KOCD8) cells 7 weeks post-transfer. **(B)** The ratio of the colon/BW in the Rag KO recipients at week 7 post-transfer. **(C)** Representative sections of colonic tissue from CTRL (score = 0), CD4 only (score = 4), CD4 + WTCD8 (score = 6), and CD4 + KOCD8 (score = 6). Colonic samples were stained with H&E and are shown at 10× magnification; scale bar = 50 μm. **(D)** The isotype controls and intracellular staining for IFN-γ and IL-17A in CD8^+^ T cells in the IEL from Rag KO mice recipients of CD4 + WTCD8 or CD4 + KOCD8 T cells. **(E)** Total IFN-γ and IL-17A in cells from Rag KO recipients of CD4 + WTCD8 or CD4 + KOCD8 T cells. Grey histograms are isotype controls. Data is from n = 6-8 mice per group and the values represent the mean of three independent experiments ± SEM. ANOVA (A, B, D) and Student’s t-tests (E), *P <0.05, **P < 0.01, ***P < 0.001.

The effect of CD8^+^ T cells on CD4/CD45RB^high^ (naïve CD4^+^) T cell induced colitis in Rag KO recipients was examined. Rag KO mice were injected with 10^6^ sorted CD8^+^ T cells from CD45.2^+^ VDR KO or CD45.2^+^ WT mice. The next day, the same Rag KO mice were injected with sorted 4 × 10^5^ WT naïve CD45.1^+^/CD4 T cells to induce colitis. Rag KO recipients of only naïve CD4 T cells (CD4 only) lost 10% of their original BW, and weighed significantly less than the CTRL mice at 7 wks post-transfer (Figure 2A). Weight loss in the Rag KO recipients of naïve WT CD4 cells plus WT CD8 T cells (CD4 + WTCD8) was no different from the recipients of CD4 only T cells (Figure 2A). Rag KO recipients of naïve WT CD4 plus VDR KO CD8 T cells (CD4 + KOCD8) lost 20% of their original BW, which was significantly more weight loss than any other group (Figure 2A). The colon/BW% was lowest in the CTRL, intermediate in the CD4 only and CD4 + WTCD8 groups and highest in the CD4 + KOCD8 group (Figure 2B). The histopathology scores from Rag KO recipients of CD4 + KOCD8 T cells were 6.5 ± 0.4, which was significantly higher than the scores for all other groups (Figure 2C and data not shown). CD8^+^ T cells from VDR KO mice produced twice as much IFN-γ and IL-17A than WT CD8^+^ T in the IEL of Rag KO recipients (Figure 2D). In addition, the Rag KO recipients of CD4 + KOCD8 T cells had significantly more total IFN-γ and IL-17A producing cells in the IEL than recipients of CD4 + WTCD8 T cells (Figure 2E). The same mice from Figure 2 were used to measure the expression of *Il-17A* and *Ifn-γ* mRNA (Additional file 3: Figure S3). *Il-17A* and *Ifn-γ* mRNA expression was higher in both the SI and colon of the Rag KO recipients of CD4 + KOCD8 T cells than the Rag KO recipients of CD4 + WTCD8 T cells (Additional file 3: Figure S3). Rag KO recipients of CD8^+^ T cells from VDR KO mice had more IFN-γ and IL-17A in the SI and colon that corresponded to the increased severity of naïve CD4^+^ T cell induced colitis.

### Increased CD8^+^, but reduced naive CD4^+^ T cells in VDR KO cell recipients

**Figure 3:**
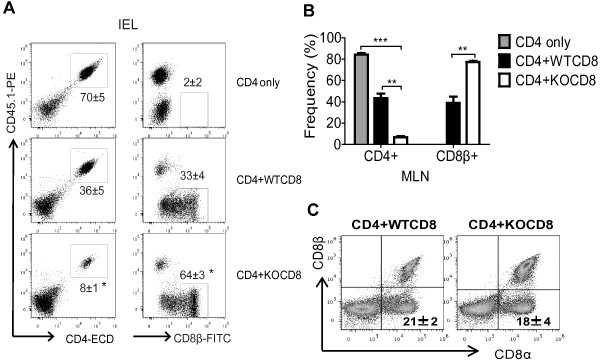
**Frequencies of CD4**^**+ **^**and CD8**^**+ **^**T cells following CD4/CD8 co-transfer. (A)** Dot plots show the frequencies of the reconstituted CD4/CD45.1^+^ and CD8β/CD45.1^−^ T cells in the IEL at week 7 post-transfer for CD4 only, CD4 + WTCD8 and CD4 + KOCD8 Rag KO recipients (mice in Figure 2). **(B)** Bar charts showing the frequency of the reconstituted CD4/CD45.1^+^ and CD8β/CD45.1^−^ T cells in the MLN. **(C)** Dot plots showing the frequency of T cells in the Rag KO recipients that expressed CD8αα in the IEL. Data is from n = 6-8 mice per group and the values represent the mean of three independent experiments ± SEM. ANOVA (A, B), or Student’s *t*-test (C), *P < 0.05, **P < 0.01, ***P < 0.001.

The MLN and IEL of the SI were used to determine the origin of T cells in the gastrointestinal tract of the Rag KO recipients. The MLN and IEL were used since these tissues normally have high frequencies of T cells and can demonstrate the extent of immune reconstitution of Rag KO mice following T cell transfer. 70 ± 5% of the IEL in Rag KO recipients of only CD4 cells were the donor CD4/CD45.1^+^ T cells (Figure 3A). The remaining cells in the IEL (30%) were the resident Rag KO IEL cells (innate immune cells) that did not express CD45.1 or CD8β. In the Rag KO recipients of CD4 + WTCD8 cells, 36 ± 5% of the IEL were CD4^+^ cells (CD45.1^+^) and 33 ± 4% were CD8αβ (CD8β^+^/CD45.1^−^) T cells such that together 69% of the IEL in the recipient were T cells (Figure 3A). The Rag KO recipients of the CD4 + KOCD8 cells had fewer CD4^+^ cells (8 ± 1%) and more CD8αβ cells (64 ± 5%) than the other groups of mice (72% total T cells, Figure 3A). The reconstitution of the MLN mirrored the IEL in that the recipients of CD4 + WTCD8 cells had approximately equal representation of CD4 and CD8αβ cells in the MLN while the mice that received CD4 + KOCD8 cells had significantly more CD8^+^ than CD4^+^ T cells in the MLN (Figure 3B). There were no differences in the total frequency of CD8αα^+^ expressing T cells (18-21% of the T cells) in the IEL of the Rag KO recipients of CD4 + WTCD8 or CD4 + KOCD8 T cells (Figure 3C). Significantly more CD8^+^ T cells were recovered in the MLN and IEL of CD4 + KOCD8 than CD4 + WTCD8 T cell transferred Rag KO recipient mice.

### Increased proliferation of VDR KO CD8^+^ T cells *in vivo*

**Figure 4:**
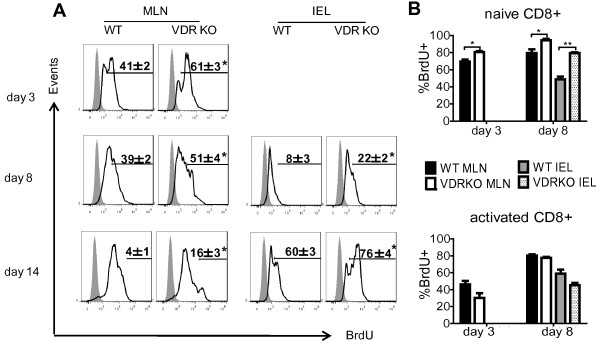
**Proliferation of CD8**^**+ **^**T cells *in vivo*. (A)** Histograms showing BrdU incorporation in the MLN or IEL following transfer of sorted 10^6^ WT or VDR KO CD8^+^ T cells into Rag KO recipients from 3 to 14 days post-transfer. Shaded histograms showed the BrdU isotype control staining, and open histograms were BrdU staining. **(B)** Different CD8^+^ subsets were evaluated for BrdU incorporation and graphed at day 3 and day 8 post-transfer. Naïve CD8^+^ T cells are CD62L^high^CD44^low^, and activated CD8^+^ T cells are CD62L^low^CD44^high^. Data is from n = 6-8 mice per group and the values represent the mean of two independent experiments ± SEM. ANOVA, *P < 0.05, **P < 0.01.

Based on the finding of increased numbers (Figure 3) of VDR KO CD8 cells in the IEL and MLN following co-transfer to Rag KO mice; additional experiments were done to determine whether VDR KO CD8 T cells proliferate more rapidly than WT CD8 T cells. Sorted CD8^+^ T cells were detectable in the MLN of Rag KO recipients on d3 following transfer (Figure 4A). Conversely, there were no T cells found in the IEL after 3 days (Figure 4A). At d3 in the MLN most of the CD8^+^ T cells from either WT or VDR KO donors were undergoing division (88 ± 2% incorporated BrdU). There were two distinct populations of BrdU^+^ cells in the MLN; one with low-mean fluorescence intensity (MFI, few rounds of proliferation) and the other one with high-MFI (Figure 4A, multiple rounds of proliferation). 61 ± 3% of VDR KO CD8^+^ T cells were of the BrdU high-MFI phenotype and only 41 ± 2% of the WT CD8^+^ T cells were BrdU high-MFI (Figure 4A). On d8 there were also more high-MFI profile VDR KO CD8^+^ T cells (51 ± 4%) than WT CD8^+^ T cells (39 ± 2%, Figure 4A). By day 14 in the MLN there was a small population (16 ± 3%) of BrdU very high-MFI VDR KO CD8^+^ T cells and significantly less of these cells in the WT CD8^+^ T recipients (4 ± 1%, Figure 4A). CD8^+^ T cells appeared in the IEL on d8 (Figure 4A). Significantly fewer cells were BrdU high-MFI in the IEL of Rag KO recipients of WT CD8^+^ T cells (8 ± 3%) than VDR KO CD8^+^ T cells (22 ± 2%, Figure 4A). There were also two distinct peaks of BrdU incorporation evident at d14 in the IEL samples and again the high-MFI peak was over represented in the recipients of VDR KO CD8^+^ T cells (Figure 4A). Total VDR KO CD8^+^ T cells were proliferating faster than WT CD8^+^ T cells *in vivo*.

To identify which T cell subsets were undergoing rapid proliferation in the Rag KO recipients, the BrdU MFI-high cells from Figure 4A were phenotyped. Naïve CD8^+^ T cells (CD62L^high^CD44^low^) from VDR KO mice proliferated more than their WT counterparts in the MLN and IEL on d3 and d8 post-transfer (Figure 4B). Conversely, activated CD8^+^ T cells (CD62L^low^CD44^high^) from VDR KO mice proliferated at the same rate (differences were not significant) as activated cells from WT mice post-transfer (Figure 4B). More naïve VDR KO CD8^+^ T cells than naïve WT CD8^+^ T cells were proliferating rapidly *in vivo*.

### Increased proliferation of VDR KO CD8^+^ T cells *in vitro*

**Figure 5:**
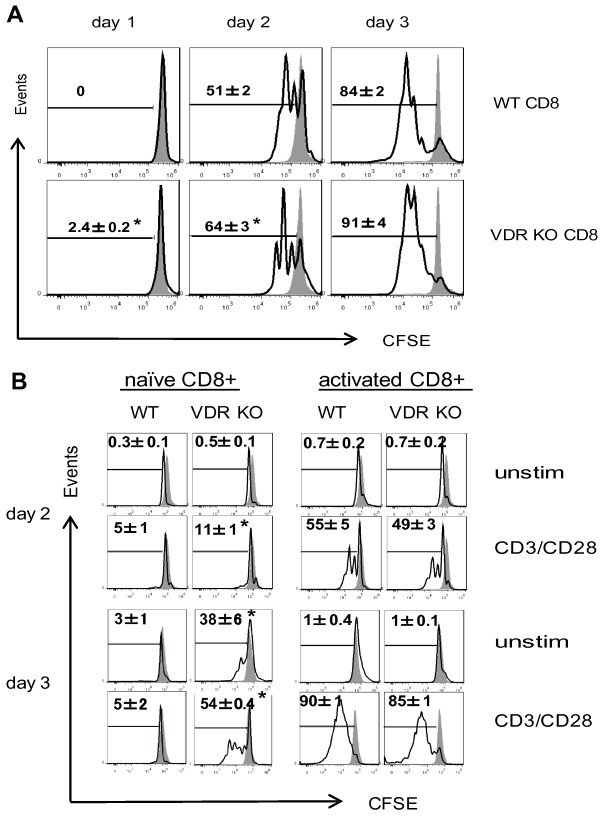
**Proliferation of CD8**^**+ **^**T cells *in vitro*. (A)** Histograms show CFSE dilution induced following *in vitro* proliferation of sorted WT and VDR KO CD8^+^ T cells in response to CD3/CD28 stimulation. The shaded histograms show the CFSE stained T cells prior to stimulation with CD3/CD28, and open histograms are CFSE dilution following stimulation. Values are mean ± SEM of the frequency of T cells that have divided and diluted the CFSE label. **(B)** The CFSE labeled T cells in panel A were evaluated for CD8 subsets with or without (unstim) CD3/CD28 stimulation. Naïve CD8^+^ T cells are CD62L^high^CD44^low^, and activated CD8^+^ T cells are CD62L^low^CD44^high^. The shaded histograms are the CFSE staining at the beginning of the culture, and open histograms are CFSE dilution. Values are the mean of two independent experiments ± SEM. ANOVA, *P < 0.05.

Proliferation of the CD8^+^ T cells from VDR KO and WT mice was also tested *in vitro*. Even though there was very little proliferation detected in the CD3/CD28 stimulated cultures at d1, VDR KO CD8^+^ T cells had 2.4 ± 0.2% of the cells proliferating while none of the WT CD8^+^ T cells were proliferating (Figure 5A). On d2 there were two rounds of proliferation evident in the WT CD8^+^ T cell cultures and three rounds of proliferation in the VDR KO CD8^+^ T cell cultures (Figure 5A). In addition, there were significantly more VDR KO CD8^+^ T cells proliferating (64 ± 3%) compared to WT CD8^+^ T cells (51 ± 2%) at d2. By d3 almost all of the cells in both cultures had proliferated and there were no longer significant differences in the frequency of CD8^+^ T cells that had undergone proliferation (Figure 5A). VDR KO CD8^+^ T cells were proliferating faster than WT CD8^+^ T cells *in vitro*.

The CFSE stained cells in panel A were also stained to look at naïve and activated cells in the cultures. On day 2 significantly more of the naïve (CD62L^high^CD44^low^) CD8^+^ T cells from VDR KO mice had proliferated in response to CD3/CD28 stimulation than WT (Figure 5B). By day 3 the naive VDR KO CD8^+^ T cells were proliferating rapidly in the presence and absence of CD3/CD28 stimulation while the naïve WT CD8^+^ T cells were not (Figure 5B). The naïve VDR KO CD8^+^ cells were dividing but not downregulating CD62L and upregulating CD44 (Figure 5B). CD8^+^ T cells with an activated phenotype (CD62L^low^CD44^high^) proliferated strongly in both the WT and VDR KO cultures at both d2 and d3 post-stimulation and there were no differences between the proliferation of the activated VDR KO and WT CD8^+^ T cells (Figure 5B). Phenotypically naïve VDR KO CD8^+^ T cells proliferated with or without stimulation but did not express activation markers as a result of proliferation.

### VDR KO CD8^+^ T cells over-produce and respond to IL-2 robustly

**Figure 6:**
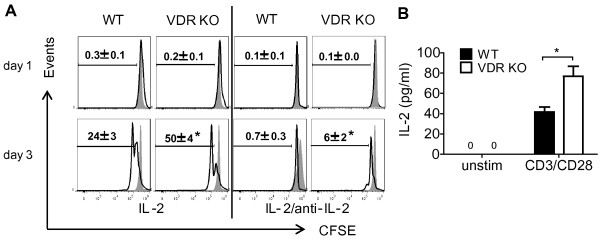
**VDR KO CD8**^**+ **^**T cells produce more and proliferate more rapidly to IL-2. (A)** Histograms show CFSE dilution following *in vitro* proliferation of sorted WT and VDR KO CD8^+^ T cells in response to IL-2 stimulation or IL-2 stimulation with IL-2 neutralizing antibodies. The shaded histograms show the CFSE stained T cells prior to stimulation. **(B)** IL-2 production was measured from sorted CD8^+^ T cells with or without (unstim) CD3/CD28 stimulation. Supernatants were collected from 3d cultures of CD8^+^ T cells, and IL-2 in the supernatant was evaluated using ELISA. Values are the means from two independent experiments ± SEM. N.D., not detected. ANOVA (A) or Student’s *t*-test (B), *P < 0.05.

The role of IL-2 in the increased proliferation of VDR KO CD8^+^ T cells was determined. CD25 expression was not different on VDR KO and WT CD8^+^ T cells (Additional file 1: Figure S1D). 3 days of culture were required in order to detect proliferation of CD8^+^ T cells in response to IL-2 (d2 data not shown, Figure 6A). 24 ± 3% of WT CD8 T cells and 50 ± 4% of VDR KO CD8^+^ T cells divided at d3 following addition of IL-2 (Figure 6A). Addition of IL-2 plus IL-2 neutralizing antibodies prevented all of the WT CD8^+^ T cell proliferation while VDR KO CD8^+^ T cells still had 6 ± 2% undergoing proliferation in the presence of neutralizing antibodies to IL-2 (Figure 6A). The supernatants from CD8^+^ T cells cultures stimulated with CD3 and CD28 antibodies were evaluated for the production of IL-2. Unstimulated VDR KO and WT CD8^+^ T cells did not produce detectable IL-2 after 3 days in culture (Figure 6B). CD3/CD28 stimulated VDR KO CD8^+^ T cells produced 2-fold more IL-2 than WT CD8^+^ T cells (Figure 6B). Overproduction of IL-2 contributes to the more rapid proliferation of CD3/CD28 activated VDR KO CD8^+^ T cells.

## Discussion

VDR KO CD8^+^ T cells proliferated more rapidly both *in vitro* and *in vivo*. The increased rate of proliferation following activation was associated with the over production of IL-2 from the CD8^+^ T cells themselves. The rapidly proliferating naïve donor VDR KO CD8^+^ T cells found residence in the MLN first and then in the IEL of the Rag KO mice where they out-competed the CD4^+^ T cells and contributed to colitis development by inducing IL-17A and IFN-γ production. In Rag KO recipients of WT CD8^+^ T cells endogenously produced 1,25(OH)_2_D_3_ slowed down the proliferation of the T cells, while the VDR KO CD8^+^ T cells could not respond to 1,25(OH)_2_D_3_. The 1,25(OH)_2_D_3_ also acted on WT CD8^+^ T cells to inhibit IL-17 and IFN- γ responses. In VDR KO mice the CD8^+^ T cells with a naïve phenotype were over-represented but their proliferation must have been controlled since VDR KO mice did not develop overt colitis symptoms [26]. VDR KO mice had normal FoxP3^+^ T regulatory cells that may have prevented the naïve CD8^+^ T cells from expanding and causing colitis [26]. Naïve CD8^+^ VDR KO T cells divided *in vitro* even in the absence of stimulation (Figure 5B). This is unique to the VDR KO CD8^+^ T cells, since naïve CD4^+^ VDR KO T cells proliferated at the same rate as naïve CD4^+^ WT T cells (unpublished data and [26]). Proliferation was not associated with upregulation of activation markers since the proliferating CD8^+^ T cells were still CD62L^high^/CD44^low^. VDR expression must be required for the quiescence of naïve CD8^+^ T cells.

Naïve and memory CD8^+^ T cells use different cytokines to regulate proliferation [27]. The proliferation of naïve T cells requires IL-7 receptor (R) α, but not IL-15; conversely, memory T cells use either IL-7Rα or IL-15 for proliferation [27]. It would be difficult to envision how this differential use of IL-7 versus IL-15 could account for the increased proliferation of predominately naïve CD8^+^ T cells. Instead it may be that developmentally the VDR is required to control the proliferation rate of the naïve CD8^+^ T cells but not the differentiated effector or memory cells. Somewhat surprisingly CD8^+^ T cells from VDR KO mice produced the same amounts of granzyme B and IFN-γ as WT CD8^+^ T cells (data not shown). Instead, CD8^+^ T cells from VDR KO mice overproduced IL-2 that may act in a paracrine manner on the CD8^+^ T cells themselves.

The original function of 1,25(OH)_2_D_3_ in the immune system was shown to be as a suppressor of mitogen induced proliferation [28]. Those early studies established that 1,25(OH)_2_D_3_ suppressed T cell proliferation in both CD4^+^ and CD8^+^ by blocking transition of the cycling cells from early G1 to late G1 [29,30]. In addition, IL-2 was shown to be a target of 1,25(OH)_2_D_3_ treatments and 1,25(OH)_2_D_3_ inhibited production of IL-2 [31]. The inhibition of IL-2 expression was due to the direct interaction of the VDR/1,25(OH)_2_D_3_ complex that blocked the binding of the NFATp/AP1 complex to the IL-2 promoter [32]. The effects of 1,25(OH)_2_D_3_ on IL-2 contributed to the suppression of T cell proliferation by 1,25(OH)_2_D_3_ but proliferation of 1,25(OH)_2_D_3_ treated T cells was only partially reversed by exogenous IL-2 addition [31]. Administration of exogenous IL-2 can increase the frequency of CD8^+^ memory cells [33,34]. Autocrine IL-2 secretion by memory CD8^+^ T cells has been shown to be a critical mechanism to maintain the CD8^+^ T cells [35]. The data in the VDR KO CD8^+^ T cells suggest that the VDR must participate as a negative co-regulator of proliferation and IL-2 production in CD8^+^ T cells. The data further suggest that expression of the VDR may be important in regulation of the memory CD8^+^ response.

Vitamin D serves as a regulator of proliferation across many different cell types. In addition to inhibiting the proliferation of T cells, 1,25(OH)_2_D_3_ inhibits the proliferation of keratinocytes, B cells, epithelial cells and several different types of cancer cells [36,37]. Topical 1,25(OH)_2_D_3_ inhibits proliferation and induces resolution of psoriasis [37]. In addition, vitamin D deficiency is associated with an increase in several different types of cancer [37]. VDR KO mice have increased epithelial cell proliferation in the gastrointestinal tract and skin [38]. More recently we have shown that in the immune system control of homeostatic proliferation and apoptosis underlie the basic mechanisms whereby vitamin D regulates TCRαβ/CD8αα and iNKT cell numbers [9,39]. Vitamin D and the VDR seem to be master regulators of proliferation across many cell types. In CD8^+^ T cells there is a selective requirement for VDR signaling to control the numbers of naïve CD8^+^ T cells in the periphery.

VDR KO CD8^+^ T cells induce colitis in the Rag KO recipients. However, the Rag KO recipients of VDR KO CD8^+^ T cells did not lose weight or develop overt symptoms of experimental IBD, suggesting that there must be some regulatory cell or cytokine being produced to limit the pathogenicity of the cells. There did not appear to be any CD28^−^ or CD122^+^ regulatory cell in the VDR KO CD8^+^ T cells. Confirming the literature the Rag KO recipients of IL-10 KO CD8^+^ T cells did not induce colitis [40]. However, IL-10/VDR DKO CD8^+^ T cells induced a fulminating form of IBD including rectal bleeding, and significant weight loss. Therefore it seems that autocrine IL-10 production by CD8^+^ T cells prevents overt colitis induced by VDR KO CD8^+^ T cells.

The VDR is critical for controlling the rate of proliferation of naïve CD8^+^ T cells and the ability of the CD8^+^ T cells to both produce and respond to IL-2. In the absence of vitamin D signaling rapidly proliferating CD8^+^ T cells accumulate in the gut and contribute to the production of IL-17 and IFN-γ. Autocrine production of IL-10 limits VDR KO CD8^+^ T cell- induced colitis. In the T cell transfer model of IBD other regulatory cells are absent that might inhibit the proliferation of the VDR KO CD8^+^ T cells to control inflammation in the gastrointestinal tract. Expression of the VDR halts the nonspecific expansion of the naïve CD8^+^ T cells. Vitamin D control of the CD8^+^ T cell response contributes to the maintenance of gastrointestinal homeostasis.

## Conclusions

In summary, VDR-deficiency results in the generation of pathogenic CD8^+^ T cells that contributes to the development of IBD. The causes of the VDR KO CD8^+^ T cell induced IBD was the rapid proliferation of CD8^+^ T cells in general and naïve CD8^+^ T cells in particular, and the overproduction and response to IL-2. Expression of the VDR prevents homeostatic proliferation of CD8^+^ T cells and vitamin D through the VDR maintains gastrointestinal homeostasis.

## Abbreviations

BW: Body weight; CTRL: Control; DKO: Double knockout; IBD: Inflammatory bowel disease; IEL: Intraepithelial lymphocyte; KO: Knockout; MFI: Mean fluorescence intensity; SI: Small intestine; VDR: Vitamin D receptor; WT: Wild type.

## Competing interests

The authors declare that they have no competing interests.

## Authors’ contributions

JC, DB and MTC designed the research; JC and DB conducted the research; JC, DB and MTC analyzed the research; JC and MTC wrote the manuscript. MTC had primary responsibility for final content. All authors read and approved the final manuscript.

## Supplementary Material

Additional file 1: Figure S1 (A) CD8+ and CD4+column purification was followed by cell sorting. Histograms show the frequencies of CD8+ T cell subsets before (pre-sort) and after (post-sort) sorting of splenocytes from WT and VDR KO mice. (B) CD4+CD45RB^high^ pre-sort and post-sort populations. The purity of the CD8+ and the CD4+CD45RB^high^ T cells was >99%. (C) Dot plots show the single staining for PE-conjugated CD45.1, FITC-conjugated CD8β, and isotype controls for both PE and FITC staining in the IEL for Figure 3A. (D) Lymphocytes were first gated on TCRβ+ cells. Bar graphs shows the frequency of CD25+ T cells within the CD8+ population. Values are the mean ± SEM of 8-10 mice per group. Student’s t-tests, n.s., not significant. Click here for file

Additional file 2: Figure S2 (A) Forward and side scatter of splenic lymphocytes. CD8β+ cells were gated on and stained for CD28, CD122 and isotype controls. (B) Forward and side scatter for the IEL and MLN. (C) Sorted CD8 cells were cultured without stimulation or with CD3/CD28 for 3 days and stained for CD8β, CD44 and CD62L antibodies. CFSE staining was analyzed in the CD44^low^/CD62L^high^ (naive) and CD44^high^/CD62L^low^ (activated) populations. Click here for file

Additional file 3: Figure S3 mRNA expression for Ifn-γ, Il-17A, and Il-10 in the (A) small intestine and (B) colon of Rag KO recipients of CD4+WTCD8 or CD4+KOCD8 (same mice as Figure 2). Data is from n=6-8 mice per group. ANOVA, *P <0.05. Click here for file

## References

[B1] Lupp C Robertson ML Wickham ME Sekirov I Champion OL Gaynor EC Finlay BB Host-Mediated Inflammation Disrupts the Intestinal Microbiota and Promotes the Overgrowth of Enterobacteriaceae Cell Host and Microbe 2007 2 119 129 10.1016/j.chom.2007.06.010 18005726

[B2] Webb AR Pilbeam C Hanafin N Holick MF An evaluation of the relative contributions of exposure to sunlight and of diet to the circulating concentrations of 25-hydroxyvitamin D in an elderly nursing home population in Boston Am J Clin Nutr 1990 51 6 1075 1081 2349922 10.1093/ajcn/51.6.1075

[B3] Ardizzone S Cassinotti A Bevilacqua M Clerici M Porro GB Vitamin D and inflammatory bowel disease Vitam Horm 2011 86 367 377 21419280 10.1016/B978-0-12-386960-9.00016-2

[B4] Pappa HM Gordon CM Saslowsky TM Zholudev A Horr B Shih MC Grand RJ Vitamin D status in children and young adults with inflammatory bowel disease Pediatrics 2006 118 5 1950 1961 10.1542/peds.2006-0841 17079566 PMC3205440

[B5] Froicu M Weaver V Wynn TA McDowell MA Welsh JE Cantorna MT A crucial role for the vitamin D receptor in experimental inflammatory bowel diseases Mol Endocrinol 2003 17 12 2386 2392 10.1210/me.2003-0281 14500760

[B6] Cantorna MT Vitamin D, multiple sclerosis and inflammatory bowel disease Arch Biochem Biophys 2012 523 1 103 106 10.1016/j.abb.2011.11.001 22085500 PMC3374859

[B7] Zella JB McCary LC DeLuca HF Oral administration of 1,25-dihydroxyvitamin D3 completely protects NOD mice from insulin-dependent diabetes mellitus Arch Biochem Biophys 2003 417 1 77 80 10.1016/S0003-9861(03)00338-2 12921782

[B8] Froicu M Zhu Y Cantorna MT Vitamin D receptor is required to control gastrointestinal immunity in IL-10 knockout mice Immunology 2006 117 3 310 318 10.1111/j.1365-2567.2005.02290.x 16476050 PMC1782241

[B9] Bruce D Cantorna MT Intrinsic requirement for the vitamin D receptor in the development of CD8alphaalpha-expressing T cells J Immunol 2011 186 5 2819 2825 10.4049/jimmunol.1003444 21270396 PMC3127166

[B10] Bruce D Yu S Ooi JH Cantorna MT Converging pathways lead to overproduction of IL-17 in the absence of vitamin D signaling Int Immunol 2011 23 8 519 528 10.1093/intimm/dxr045 21697289 PMC3139478

[B11] Mayne CG Spanier JA Relland LM Williams CB Hayes CE 1,25-Dihydroxyvitamin D3 acts directly on the T lymphocyte vitamin D receptor to inhibit experimental autoimmune encephalomyelitis Eur J Immunol 2011 41 3 822 832 10.1002/eji.201040632 21287548

[B12] Mahon BD Wittke A Weaver V Cantorna MT The targets of vitamin D depend on the differentiation and activation status of CD4 positive T cells J Cell Biochem 2003 89 5 922 932 10.1002/jcb.10580 12874827

[B13] Thien R Baier K Pietschmann P Peterlik M Willheim M Interactions of 1 alpha,25-dihydroxyvitamin D3 with IL-12 and IL-4 on cytokine expression of human T lymphocytes J Allergy Clin Immunol 2005 116 3 683 689 10.1016/j.jaci.2005.05.013 16159643

[B14] Yu S Cantorna MT The vitamin D receptor is required for iNKT cell development Proc Natl Acad Sci USA 2008 105 13 5207 5212 10.1073/pnas.0711558105 18364394 PMC2278204

[B15] Barrat FJ Cua DJ Boonstra A Richards DF Crain C Savelkoul HF de Waal-Malefyt R Coffman RL Hawrylowicz CM O’Garra A In vitro generation of interleukin 10-producing regulatory CD4(+) T cells is induced by immunosuppressive drugs and inhibited by T helper type 1 (Th1)- and Th2-inducing cytokines J Exp Med 2002 195 5 603 616 10.1084/jem.20011629 11877483 PMC2193760

[B16] Gregori S Giarratana N Smiroldo S Uskokovic M Adorini L A 1alpha,25-dihydroxyvitamin D(3) analog enhances regulatory T-cells and arrests autoimmune diabetes in NOD mice Diabetes 2002 51 5 1367 1374 10.2337/diabetes.51.5.1367 11978632

[B17] Veldman CM Cantorna MT DeLuca HF Expression of 1,25-dihydroxyvitamin D(3) receptor in the immune system Arch Biochem Biophys 2000 374 2 334 338 10.1006/abbi.1999.1605 10666315

[B18] Steinhoff U Brinkmann V Klemm U Aichele P Seiler P Brandt U Bland PW Prinz I Zugel U Kaufmann SHE Autoimmune intestinal pathology induced by hsp60-specific CD8 T cells Immunity 1999 11 3 349 358 10.1016/S1074-7613(00)80110-7 10514013

[B19] Wiesel M Oxenius A From crucial to negligible: functional CD8(+) T-cell responses and their dependence on CD4(+) T-cell help Eur J Immunol 2012 42 5 1080 1088 10.1002/eji.201142205 22539281

[B20] Filaci G Fravega M Negrini S Procopio F Fenoglio D Rizzi M Brenci S Contini P Olive D Ghio M Nonantigen specific CD8(+) T suppressor lymphocytes originate from CD8(+)CD28(-) T cells and inhibit both T-cell proliferation and CTL function Hum Immunol 2004 65 2 142 156 10.1016/j.humimm.2003.12.001 14969769

[B21] Menager-Marcq I Pomie C Romagnoli P van Meerwijk JPM CD8(+)CD28(-) regulatory T lymphocytes prevent experimental inflammatory bowel disease in mice Gastroenterology 2006 131 6 1775 1785 10.1053/j.gastro.2006.09.008 17087950 PMC1950262

[B22] Shi Z Okuno Y Rifa’i M Endharti AT Akane K Isobe KI Suzuki H Human CD8(+) CXCR3(+) T cells have the same function as murine CD8(+) CD122(+) Treg Eur J Immunol 2009 39 8 2106 2119 10.1002/eji.200939314 19609979

[B23] Endharti AT Okuno Y Shi Z Misawa N Toyokuni S Ito M Isobe K Suzuki H CD8(+)CD122(+) Regulatory T Cells (Tregs) and CD4(+) Tregs Cooperatively Prevent and Cure CD4(+) Cell-Induced Colitis J Immunol 2011 186 1 41 52 10.4049/jimmunol.1000800 21098236

[B24] Poussier P Ning T Banerjee D Julius M A unique subset of self-specific intraintestinal T cells maintains gut integrity J Exp Med 2002 195 11 1491 1497 10.1084/jem.20011793 12045247 PMC2193537

[B25] Das G Augustine MM Das J Bottomly K Ray P Ray A An important regulatory role for CD4 + CD8 alpha alpha T cells in the intestinal epithelial layer in the prevention of inflammatory bowel disease Proc Natl Acad Sci USA 2003 100 9 5324 5329 10.1073/pnas.0831037100 12695566 PMC154344

[B26] Yu S Bruce D Froicu M Weaver V Cantorna MT Failure of T cell homing, reduced CD4/CD8alphaalpha intraepithelial lymphocytes, and inflammation in the gut of vitamin D receptor KO mice Proc Natl Acad Sci USA 2008 105 52 20834 20839 10.1073/pnas.0808700106 19095793 PMC2634903

[B27] Goldrath AW Sivakumar PV Glaccum M Kennedy MK Bevan MJ Benoist C Mathis D Butz EA Cytokine requirements for acute and basal homeostatic proliferation of naive and memory CD8(+) T cells J Exp Med 2002 195 12 1515 1522 10.1084/jem.20020033 12070279 PMC2193554

[B28] Tsoukas CD Provvedini DM Manolagas SC 1,25-dihydroxyvitamin D3: a novel immunoregulatory hormone Science 1984 224 4656 1438 1440 10.1126/science.6427926 6427926

[B29] Rigby WF Noelle RJ Krause K Fanger MW The effects of 1,25-dihydroxyvitamin D3 on human T lymphocyte activation and proliferation: a cell cycle analysis J Immunol 1985 135 4 2279 2286 2993410

[B30] Rigby WF Yirinec B Oldershaw RL Fanger MW Comparison of the effects of 1,25-dihydroxyvitamin D3 on T lymphocyte subpopulations Eur J Immunol 1987 17 4 563 566 10.1002/eji.1830170420 3106071

[B31] Rigby WF Stacy T Fanger MW Inhibition of T lymphocyte mitogenesis by 1,25-dihydroxyvitamin D3 (calcitriol) J Clin Invest 1984 74 4 1451 1455 10.1172/JCI111557 6332829 PMC425314

[B32] Alroy I Towers TL Freedman LP Transcriptional repression of the interleukin-2 gene by vitamin D3: direct inhibition of NFATp/AP-1 complex formation by a nuclear hormone receptor Mol Cell Biol 1995 15 10 5789 5799 7565732 10.1128/mcb.15.10.5789 PMC230831

[B33] Boyman O Kovar M Rubinstein MP Surh CD Sprent J Selective stimulation of T cell subsets with antibody-cytokine immune complexes Science 2006 311 5769 1924 1927 10.1126/science.1122927 16484453

[B34] Blattman JN Grayson JM Wherry EJ Kaech SM Smith KA Ahmed R Therapeutic use of IL-2 to enhance antiviral T-cell responses in vivo Nat Med 2003 9 5 540 547 10.1038/nm866 12692546

[B35] Feau S Arens R Togher S Schoenberger SP Autocrine IL-2 is required for secondary population expansion of CD8(+) memory T cells Nat Immunol 2011 12 9 908 913 10.1038/ni.2079 21804558 PMC3388550

[B36] Lemire JM Adams JS Sakai R Jordan SC 1 alpha,25-dihydroxyvitamin D3 suppresses proliferation and immunoglobulin production by normal human peripheral blood mononuclear cells J Clin Invest 1984 74 2 657 661 10.1172/JCI111465 6611355 PMC370520

[B37] Carlberg C Molnar F Current status of vitamin D signaling and its therapeutic applications Curr Top Med Chem 2012 12 6 528 547 10.2174/156802612799436623 22242854

[B38] Welsh J Cellular and molecular effects of vitamin D on carcinogenesis Arch Biochem Biophys 2012 523 1 107 114 10.1016/j.abb.2011.10.019 22085499 PMC3295909

[B39] Yu S Cantorna MT Epigenetic reduction in invariant NKT cells following in utero vitamin D deficiency in mice J Immunol 2011 186 3 1384 1390 10.4049/jimmunol.1002545 21191070 PMC3127168

[B40] Davidson NJ Leach MW Fort MM Thompson-Snipes L Kuhn R Muller W Berg DJ Rennick DM T helper cell 1-type CD4+ T cells, but not B cells, mediate colitis in interleukin 10-deficient mice J Exp Med 1996 184 1 241 251 10.1084/jem.184.1.241 8691138 PMC2192682

