# Supplementary material for: Vitamin D receptor expression controls proliferation of naïve CD8+ T cells and development of CD8 mediated gastrointestinal inflammation
Source: BMC Immunol. 2014 Feb 7;15:6. doi: 10.1186/1471-2172-15-6 (PMC3923390; doi:10.1186/1471-2172-15-6)
Supplement: Additional file 1: Figure S1 — (A) CD8+ and CD4+column purification was followed by cell sorting. Histograms show the frequencies of CD8+ T cell subsets before (pre-sort) and after (post-sort) sorting of splenocytes from WT and VDR KO mice. (B) CD4+CD45RBhigh pre-sort and post-sort populations. The purity of the CD8+ and the CD4+CD45RBhigh T cells was >99%. (C) Dot plots show the single staining for PE-conjugated CD45.1, FITC-conjugated CD8β, and isotype controls for both PE and FITC staining in the IEL for Figure 3A. (D) Lymphocytes were first gated on TCRβ+ cells. Bar graphs shows the frequency of CD25+ T cells within the CD8+ population. Values are the mean ± SEM of 8-10 mice per group. Student’s t-tests, n.s., not significant. [file 1471-2172-15-6-S1.pdf]

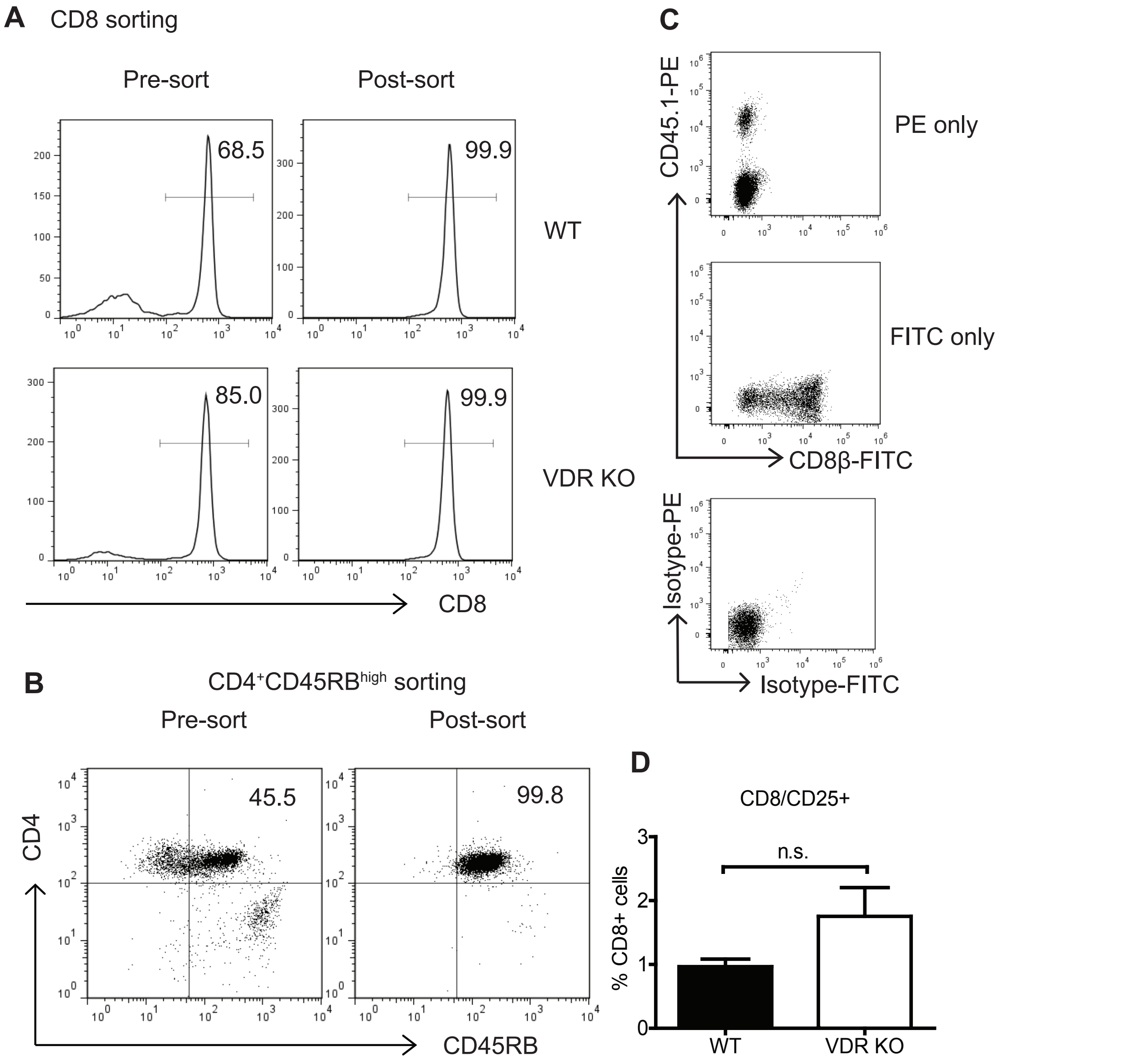

Supplementary Figure 1. (A) CD8^{+} and CD4^{+}column purification was followed by cell sorting. Histograms show the frequencies of CD8^{+} T cell subsets before (pre-sort) and after (post-sort) sorting of splenocytes from WT and VDR KO mice. (B) CD4^{+}CD45RB^{high} pre-sort and post-sort populations. The purity of the CD8^{+} and the CD4^{+}CD45RB^{high} T cells was >99%. (C) Dot plots show the single staining for PE-conjugated CD45.1, FITC-conjugated CD8β, and isotype controls for both PE and FITC staining in the IEL for Fig. 3A. (D) Lymphocytes were first gated on TCRβ^{+} cells.Bar graphs shows the frequency of CD25^{+} T cells within the CD8^{+} population. Values are the mean ± SEM of 8-10 mice per group. Student's t-tests, n.s., not significant.
