# Supplementary material for: Vitamin D receptor expression controls proliferation of naïve CD8+ T cells and development of CD8 mediated gastrointestinal inflammation
Source: BMC Immunol. 2014 Feb 7;15:6. doi: 10.1186/1471-2172-15-6 (PMC3923390; doi:10.1186/1471-2172-15-6)
Supplement: Additional file 2: Figure S2 — (A) Forward and side scatter of splenic lymphocytes. CD8β+ cells were gated on and stained for CD28, CD122 and isotype controls. (B) Forward and side scatter for the IEL and MLN. (C) Sorted CD8 cells were cultured without stimulation or with CD3/CD28 for 3 days and stained for CD8β, CD44 and CD62L antibodies. CFSE staining was analyzed in the CD44low/CD62Lhigh (naive) and CD44high/CD62Llow (activated) populations. [file 1471-2172-15-6-S2.pdf]

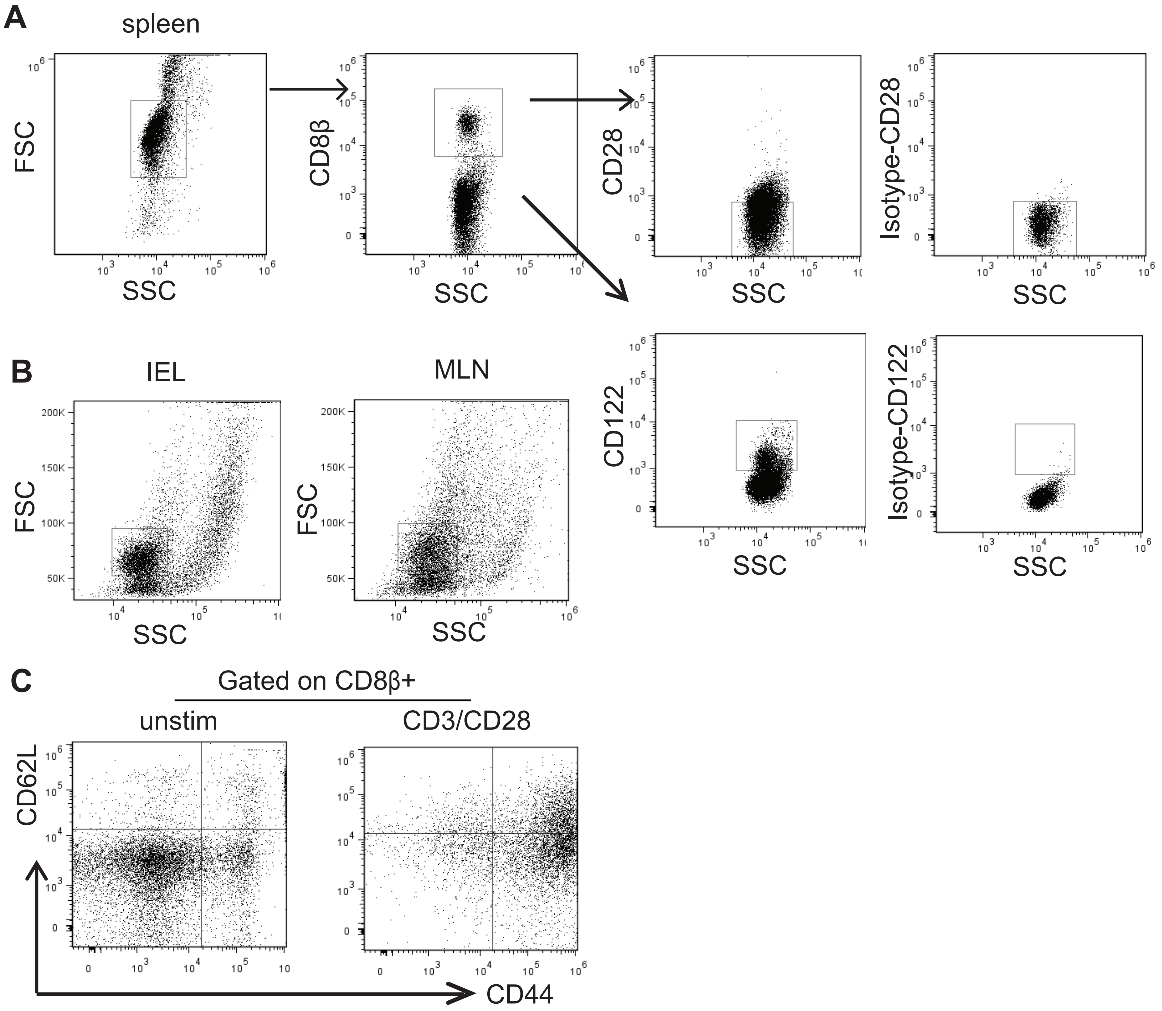

Supplementary Figure 2. (A) Forward and side scatter of splenic lymphocytes. CD8β^{+} cells were gated on and stained for CD28, CD122 and isotype controls. (B) Forward and side scatter for the IEL and MLN. (C) Sorted CD8 cells were cultured without stimulation or with CD3/CD28 for 3 days and stained for CD8β, CD44 and CD62L antibodies. CFSE staining was analyzed in the CD44^{low}/CD62L^{high} (naive) and CD44^{high}/CD62L^{low} (activated) populations.
