# Supplementary material for: Vitamin D receptor expression controls proliferation of naïve CD8+ T cells and development of CD8 mediated gastrointestinal inflammation
Source: BMC Immunol. 2014 Feb 7;15:6. doi: 10.1186/1471-2172-15-6 (PMC3923390; doi:10.1186/1471-2172-15-6)
Supplement: Additional file 3: Figure S3 — mRNA expression for Ifn-γ, Il-17A, and Il-10 in the (A) small intestine and (B) colon of Rag KO recipients of CD4+WTCD8 or CD4+KOCD8 (same mice as Figure 2). Data is from n=6-8 mice per group. ANOVA, *P <0.05. [file 1471-2172-15-6-S3.pdf]

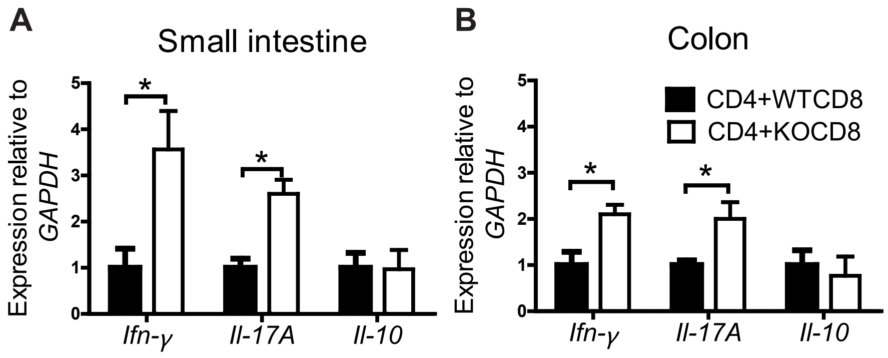


Supplemental Figure 3. mRNA expression for Ifn-γ, Il-17A, and Il-10 in the (A) small intestine and (B) colon of Rag KO recipients of CD4+WTCD8 or CD4+KOCD8 (same mice as Fig. 2). Data is from n=6-8 mice per group. ANOVA, *P <0.05.
